# *Demodex bialoviensis* sp. nov. (Acariformes, Demodecidae) a new, specific parasite of the European bison *Bison bonasus* (Artiodactyla, Bovidae)

**DOI:** 10.1016/j.ijppaw.2022.01.003

**Published:** 2022-01-13

**Authors:** Joanna N. Izdebska, Leszek Rolbiecki, Wojciech Bielecki

**Affiliations:** a Department of Invertebrate Zoology and Parasitology, Faculty of Biology, University of Gdańsk, Wita Stwosza 59, 80-308, Gdańsk, Poland; b Institute of Veterinary Medicine, Warsaw University of Life Sciences, Department of Pathology and Veterinary Diagnostics, Division of Avian Diseases, Exotic Animals and Fish, Ciszewskiego 8, 02-786, Warsaw, Poland

**Keywords:** Acariformes, Demodecidae, Artiodactyla, European bison, Mites, Wild ungulates

## Abstract

Sixteen species of parasitic mites of the family Demodecidae have been described in ungulates (Ungulatomorpha), particularly among domestic animals and livestock. Specific synhospital species have been found in seven host species. *Demodex bisonianus*, occurring in the Meibomian glands of the eyelids, was described in the European bison *Bison bonasus*. Together with *Trypanosoma wrublewskii* and *Bisonicola sedecimdecembrii*, it is one of the three known specific parasite species of European bison. The European bison is not only a rare and endangered mammal, but its biology has been shaped by its recent restitution from extinction from a small pool of individuals kept in breeding facilities. This could have been the reason for the extinction of the majority of specific parasites. The present study reports the finding of a new specific parasite species, *Demodex bialoviensis* sp. nov., associated with the nasal skin region, in European bison from the Białowieża Forest (Poland). It is the second species of *Demodex* reported in this host species, however occurring in a different location.

## Highlights

• The European bison is the largest European land mammal; despite its successful restitution, remains highly endangered.
• Only three specific parasite species have been known in the European bison, including one mite – *Demodex bisonianus.*
• Currently, another representative of Demodecidae, *Demodex bialoviensis* sp. nov. has been found in the European bison.
• *Demodex. bisonianus* occurs only in the area of the eyelids, and *D. bialoviensis* sp. nov. in the nasal skin region.

## Graphical abstract

**Figure:**
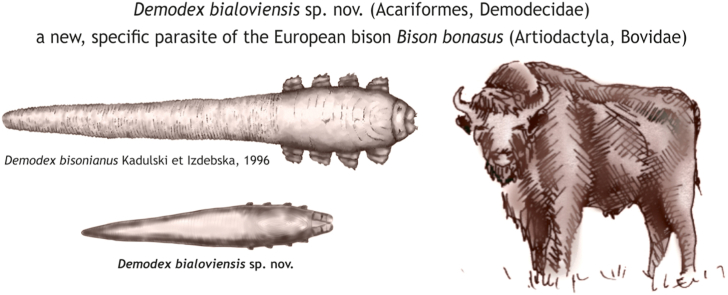

## 1 Introduction

Despite its successful restitution from extinction in the wild, the European bison *Bison bonasus* Linnaeus, 1758, the largest European land mammal and a relic of Pleistocene megafauna, remains highly endangered. The total world population is 9111 (more than half of which live in Poland and Belarus), of which 6819 are free-living (as of December 31, 2020) and the rest live in various breeding centers or zoological gardens (Raczyński, 2020). The species currently faces threats from its shrinking natural habitat, its low level of heterozygosity due to the high level of inbreeding that took place during its restitution, and its low resistance and susceptibility to disease (Pucek et al., 2004; Tokarska et al., 2011; Larsta and Krzysiak, 2019) resulting from the small gene pool of the current population. In natural conditions, the last bison of the lowland subspecies *B. b. bonasus* died in 1919 in the Białowieża Forest, and it is likely that the last mountain subspecies, Caucasian bison *B. b. caucasicus*, went extinct even earlier although, according to some sources, it survived until 1927 (Krasińska and Krasiński, 2013; Krasiński et al., 2016)*.* In 1923, it was decided to restore the species based on the individuals that had survived in breeding facilities and zoological gardens; the site of the breeding program was to be the Białowieża Forest, the natural habitat of the European bison. These efforts were rewarded with the restoration of the lowland-line and the lowland-Caucasian line bison, the latter derived from the last bison of the Caucasian mountain subspecies (Krasiński et al., 2016). As all living bison are descended from 12 individuals, with the pure lowland line bison from only seven individuals (Olech, 2009), the species is a very interesting object for parasitological research. Ungulates usually demonstrate characteristic parasitofauna formed under specific environmental conditions over the course of the long-term evolution of the host-parasite relationship. Given the artificial nature of their breeding conditions, modern bison could have lost part of this parasitofauna, and this would have been supported by the fact that the species was restored from a small number of individuals (Izdebska 2007, 2011). Hence, while 88 parasite species have been identified in modern European bison, only three species are specific for the species, and the rest are typical for cattle or acquired from cervids (Karbowiak et al., 2014). The first known specific parasite was *Trypanosoma wrublewski* Wladimiroff et Yakimoff, 1909, followed by *Bisonicola sedecimdecembrii* (Eichler, 1946) (redesc. Izdebska 2011), which is common in the host population and usually asymptomatic (Izdebska 2003, 2011). It was not until the end of the 20th century that a specific skin mite of the family Demodecidae (*Demodex bisonianus* Kadulski et Izdebska, 1996) was discovered and described; however, although it demonstrates a high prevalence, it is difficult to detect due to its hidden lifestyle (Meibomian glands of the eyelids), microscopic size and lack of signs of infestation (Izdebska, 2006). Studies of Demodecidae are problematic, especially in large ungulates, where they are often present at low densities: wild hosts rarely demonstrate disease symptoms which typically lead to detection. Among the Ungulatomorpha, 16 species of parasitic mite from the family Demodecidae have been described so far, with most being associated with domestic animals and livestock. Seven host species were found to harbor two or three synhospital species of demodecid mites (co-occurring in a single host species) specific to the host species (Izdebska and Rolbiecki, 2020). The single *D. bisonianus* species previously found in bison was discovered due to the high prevalence of infection and restricted location (around the eyes), the typical for many species of Demodecidae family (Kadulski and Izdebska, 1996; Izdebska, 2009). A few specimens of an unknown species of *Demodex* sp. were also found, but the material was too scarce and their state of preservation did not allow for taxonomic analysis and the preparation of a scientific description (Izdebska, 2006).

More recently, other specimens have been found in bison studied in the Białowieża Forest. Based on taxonomic criteria adopted for the Demodecidae family, these have been recognized as a new species of *Demodex bialoviensis* sp. nov. However, unlike *D. bisonianus*, the new species was found only in the skin of the nasal region; which is to be expected considering the great host specificity and topicality demonstrated by other synhospital *Demodex* species.

## 2 Materials and methods

The research was carried out during the winter and summer eliminations of European bison in the Białowieża Forest in the years 2011–2012. During these programs, twelve (6 from July 2011 and 6 from February 2012) bison were examined for demodecid mites.

The skin mites were recovered by host skin fragment digestion (Izdebska, 2004), with modifications to suit the examined host. Skin fragments of 3 cm^2^ were examined from several body regions, including the head (around the eyes, nose, area of vibrissae, lips, chin, cheeks, vertex), neck, abdomen, back, limbs and genital-anal area. Skin samples were preserved in 70% ethanol and subjected to digestion in 10% KOH solution; the obtained samples were decanted (the examination of 1 cm^2^ of skin was equal to that of approximately 100 wet preparations) and examined using phase-contrast microscopy (Nikon Eclipse 50i). Specimens were placed in polyvinyl-lactophenol solution. All measurements are given in micrometers. The following measurements were taken: total body length = length of gnathosoma, podosoma and opisthosoma; gnathosomal width = width at base; podosomal and opisthosomal width = maximum width.

Specimen depositories are cited using the abbreviation UGDIZP, University of Gdańsk, Department of Invertebrate Zoology and Parasitology, Gdańsk, Poland (Zhang, 2018). The description of the species adopted the nomenclature commonly used for the family Demodecidae (Nutting, 1976) and was completed with the nomenclature proposed by Bochkov (2008) for the superfamily Cheyletoidea, and by Izdebska and Rolbiecki (2016). The scientific and common names of the hosts follow Wilson and Reeder (2005) and the Integrated Taxonomic Information System (2021).

The prevalence and density were calculated to determine the level of host infection (Bush et al., 1997).

This paper and the nomenclatural act it contains have been registered in Zoobank (www.zoobank.org), the official register of the International Commission on Zoological Nomenclature. The LSID (Life Science Identifier) number of the publication is: urn:lsid:zoobank.org:pub:9CD36429-B00D-48CD-A86A-99A5E5473AAE.

## 3 Results

### 3.1 Descriptions

FEMALE (n = 33 and 1 holotype): Body slender, elongated, spindle-shaped, with distinctly separated gnathosoma, 239 (200–268) long and wide 35 (30–40) (holotype, 250 × 39). Gnathosoma rectangular (length close or greater to width at base); on dorsal surface in central part of basal (coxal) segment, pair of wedge-shaped supracoxal spines (setae *elc.p*) present, ca. 4.0 long (holotype, 4.0), directed medially, slightly oblique. Palps 3-segmented, terminating in three bifurcated spines (one large ca. 3.0, one medium and one small) on tibio-tarsus; conical setae *v’’F* near external edge of middle segment (trochanter-femur-tarsus) present. On ventral surface, horseshoe-shaped pharyngeal bulb with pair of conical subgnathosomal setae (setae *n*) situated anterior on both sides. Podosoma rectangular; four pairs of short legs, with coxa integrated into ventral idiosomal wall and five free, overlapping segments (trochanter-tarsus); two forked claws, ca. 5.0 long (holotype, 5.0), with large, pointed subterminal spur on each tarsus. Epimeral plates (coxal fields) distinctly sclerotized; pair I triangular, II-IV trapezoidal, posterior edges of pair IV deeply arched with vulva inside. On the dorsal side of podosoma podosomal shield present, with distinct vertical striation, reaching level of legs III; posterior edge convex. Opisthosoma constitutes 65% (60–69%) of body length (holotype, 65%); conical, pointed or slightly rounded at end. Whole opisthosoma distinctly annulated; annulation reaches level of legs III dorsally; annuli relatively wide ca. 1.5–2.0. Opisthosomal organ tubular-shape (ca. 10 in length) and is located in posterior part of opisthosoma; its posterior edge is located ca. 20 from end of opisthosoma. Vulva 12 (10–17) long (holotype, 13).

MALE (n = 12): Slightly larger and slender than female, 176 (158–198) long, 31 (30–35) wide. Gnathosoma shape similar to female, but smaller. Pharyngeal bulb and morphological details of gnathosoma similar to those in female, but supracoxal spines smaller (ca. 2 in length). Shape of podosoma and legs similar to those in female, but claws smaller (4 in length), and posterior edge of epimeral plate IV without archwise. Opisthosoma constitutes 62% (57–65%) of body length; whole opisthosoma, similar to female, distinctly annulated; annuli relatively wide at ca. 1.5–2.0. Opisthosomal organ similar to female, but smaller, its posterior edge is located ca. 15 from end of opisthosoma. Aedeagus 21 (18–29) long, on dorsal surface, located between epimeral plates II and IV. Genital opening located on dorsal surface, slightly above on border between epimeral plates I and II.

DEUTONYMPH (n = 5): Body elongated, club shaped, strongly tapering towards end, 202 (188–218) long, 36 (35–38) wide. Gnathosoma rectangular (width similar to or longer than width at base), smaller than in adults. Supracoxal spines conical, ca. 1 long, located on dorsal side at external edges of gnathosoma. Pharyngeal bulb round, subgnathosomal setae not visible. Palps 3-segmented, terminating in three small spines on tibio-tarsus. Four pairs of small, unsegmented legs, equipment with two 3-pointed claws. Four pairs of ventral scutes (I pair smaller), located between legs in middle part of podosoma. Opisthosoma constitutes 66% (63–68%) of body length; whole opisthosoma distinctly annulated.

### 3.2 Material deposition

Female holotype (reg. no. UGDIZPBBbDDb11f), 33 female paratypes (reg. no. UGDIZPBBbDDb01f−10f, UGDIZPBBbDDb12f−34f), 12 male paratypes (reg. no. UGDIZPBBbDDb01m−12m), and five deutonymphs (reg. no. UGDIZPBBbDDb01d−05d); skin of the nasal region; host *Bison bonasus* (reg. no. MABBb01/2011, MABBb03/2011, MABBb03/2012); Białowieża Forest, Poland; July 2011 and December 2012; parasites coll. J.N. Izdebska and L. Rolbiecki; deposited within the framework of the Collection of Extant Invertebrates in Department of Invertebrate Zoology and Parasitology, University of Gdańsk, Poland.

### 3.3 Etymology

The specific epithet *bialoviensis* refers to the geographic name (Białowieża) of the host locality.

### 3.4 Infestation and location in the host

*Demodex bialoviensis* sp. nov. was noted in 3 (prevalence 25%) European bison, with a density of 1.9 per 1 cm^2^; 51 (12 males, 34 females, and 5 deutonymphs) individuals were noted. The demodecid mites were found in the nasal skin region. The observed mites did not cause any skin lesions in the examined bison.

### 3.5 Differential diagnosis

**Fig. 1:**
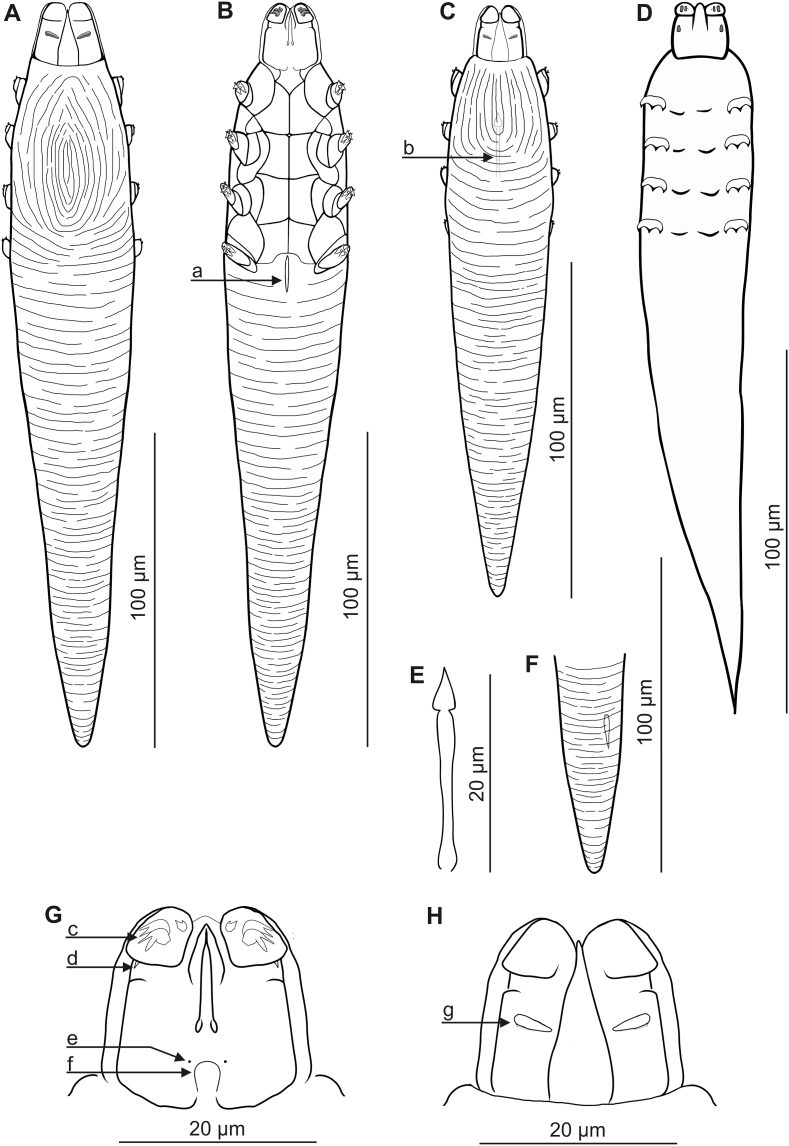
*Demodex bialoviensis* sp. nov.: A, female, dorsal view; B, female, ventral view, a. vulva; C, male, dorsal view, b. aedeagus; D, deutonypmps, ventral view; E, aedeagus; F, posterior part of opisthosoma with visible opisthosomal organ; G, gnathosoma, female, ventral view, c. spines on palps, d. seta *v”F*, e. subgnathosomal seta (seta *n*), f. pharyngeal bulb; H, gnathosoma, female, dorsal view, g. supracoxal spine (seta *elc.p*).

**Fig. 2:**
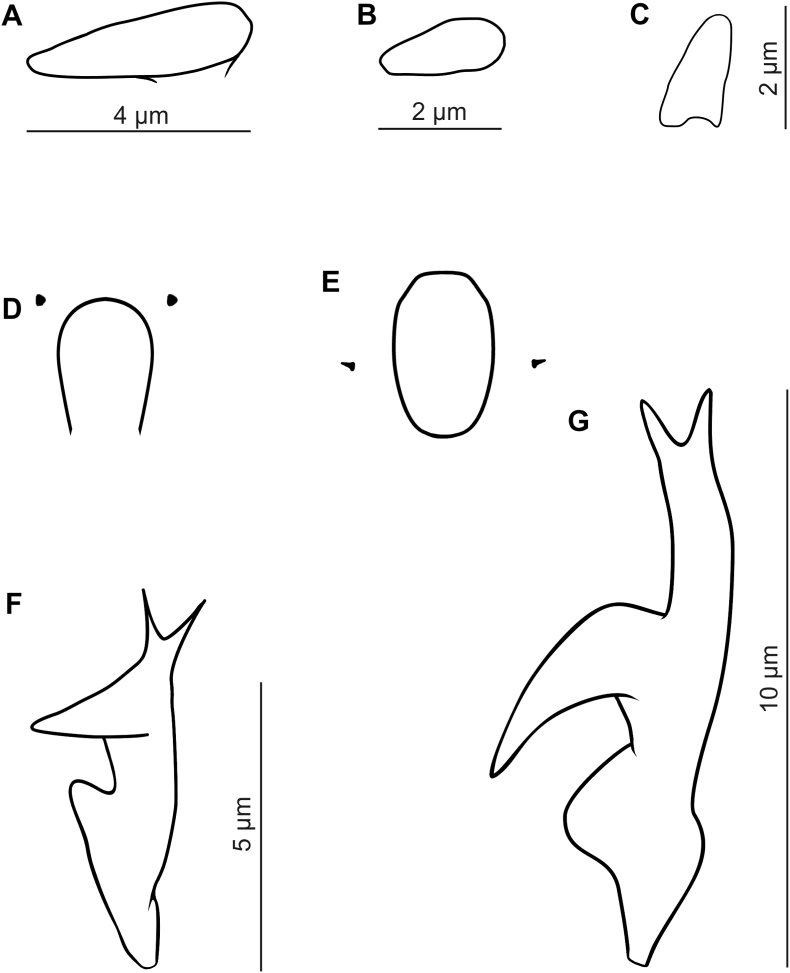
Supracoxal spines (A–C), pharyngeal bulbs with subgnathosomal setae (D–E), and claws on the leg tarsi (F and G). *Demodex bialoviensis* sp. nov., A (female), B (male), D, F; *Demodex bisonianus*, C, E, G.

**Fig. 3:**
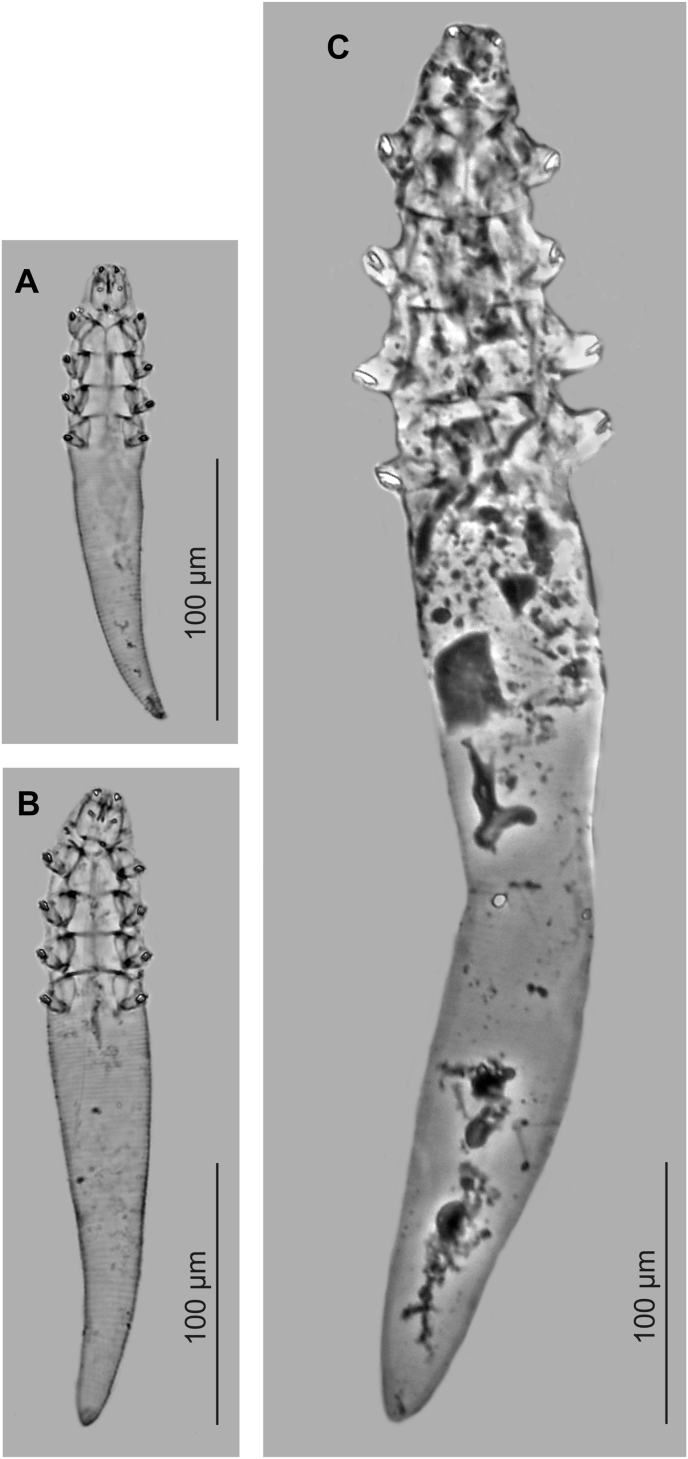
*Demodex bialoviensis* sp. nov. (A, male; B, female) and *Demodex bisonianus* (C, female), scaled.

Compared to *D. bisonianus* previously described in bison (Kadulski and Izdebska, 1996; Izdebska, unpublished data), *D. bialoviensis* sp. nov. is much smaller, with a different body shape and proportions. In addition, *D. bialoviensis* sp. nov. shows a clear sexual dimorphism: the males are much smaller (Table 1, Table 2, Fig. 1, Fig. 2, Fig. 3).

**Table 1:** Body size (micrometers) for adults and deutonymphs of *Demodex bialoviensis* sp. nov.

Morphologic features	Males (n = 12)	Females (n = 34)	Deutonymph (n = 5)
Length of gnathosoma	17 (15–20), SD 1	22 (20–26), SD 2	16 (13–20), SD 3
Width of gnathosoma (at base)	15 (13–20), SD 2	21 (17–25), SD 2	14 (11–17), SD 3
Length of podosoma	50 (45–58), SD 4	62 (55–68), SD 4	53 (50–58), SD 3
Width of podosoma	31 (30–35), SD 2	35 (30–40), SD 2	36 (35–38), SD 1
Length of opisthosoma	109 (93–125), SD 11	154 (125–178), SD 13	133 (125–145), SD 9
Width of opisthosoma	29 (25–33), SD 2	33 (30–39), SD 3	31 (30–33), SD 1
Aedeagus	21 (18–29), SD 3	–	–
Vulva	–	12 (10–17), SD 2	–
Total length of body	176 (158–198), SD 13	239 (200–268), SD 15	202 (188–218), SD 12

**Table 2:** Morphometric comparison between *Demodex bialoviensis* sp. nov. and *Demodex bisonianus*.

Feature/Species	*Demodex bialoviensis* sp. nov.		*Demodex bisonianus*	
Source	Present study		Kadulski and Izdebska (1996)	
Sex Sample size	Males (n = 12)	Females (n = 34)	Males (n = 20)	Females (n = 20)
Body total length	176 (158–198), SD 13	239 (200–268), SD 15	517, SD 23^a^	534, SD 20^a^
Body total width	31 (30–35), SD 2	35 (30–40), SD 2	63, SD 9	68, SD 9
Body length to width ratio	5.6:1 (4.9–6.4:1), SD 0.6:1	6.8:1 (5.2–7.9:1), SD 0.6:1	8.2:1^b^	7.9:1^b^
Opisthosoma length to body length ratio (%)	62 (57–65), SD 2	65 (60–69), SD 2	68^b^	69^b^
Aedeagus length	21 (18–29), SD 3	–	33, SD 7	–
Vulva length	–	12 (10–17), SD 2	–	22, SD 2

a Measurements were rounded to the nearest micrometer with respect to the original results (Kadulski and Izdebska, 1996).

b Calculated from measurements of Kadulski and Izdebska (1996).

The gnathosoma of *D. bisonianus* is trapezoidal, with a length less than the width at the base; in *D. bialoviensis* sp. nov. it is rectangular, almost square, with a length close to the width at the base. Supracoxal spines in *D. bisonianus* are relatively small, conical, situated in the anterior part of the basal segment of gnathosoma, directed vertically, while in *D. bialoviensis* sp. nov. they are relatively larger, wedge-shaped, situated in the central part of the basal segment and directed horizontally (medially and slightly oblique). On the terminal segments of the palpi, two larger spines and one small spine are present in *D. bisonianus*, and three forked spines of different sizes in *D. bialoviensis* sp. nov. Subgnathosomal setae are located on the both sides in the lower half of the pharyngeal bulb in *D. bisonianus*, and at the anterior part of the pharyngeal bulb in *D. bialoviensis* sp. nov. The legs of *D. bisonianus* are very massive, clearly projecting beyond the margin of the podosoma, and the tarsi of the legs are equipped with very large (ca. 9–10 long), strongly forked claws; in contrast, the legs of *D. bialoviensis* sp. nov. are relatively smaller, with more delicate (4–5 long) and less forked claws. The posterior edges of the IV pair of epimeral plates in *D. bisonianus* females is V-shaped, while in *D. bialoviensis* sp. nov. it is deeply arched. The aedeagus of *D. bisonianus* males is much longer, located at the level of the II-III pair of epimeral plates, while in *D. bialoviensis* sp. nov. it is shorter, stocky and located at the level of the II-IV pair of plates. The opisthosoma is narrow and long, cylindrical, rounded at the end in *D. bisonianus*, but tapers distinctly posteriorly in *D. bialoviensis* sp. nov, with a sharp or slightly rounded end. The opisthosomal organ is absent in *D. bisonianus*, but present in *D. bialoviensis*. The typical microhabitat is also different: *D. bisonianus* is found in the Meibomian glands of the eyelids, and *D. bialoviensis* sp. nov. in the nasal skin region.

In contrast, the shape of *D. bialoviensis* sp. nov. resembles that of *D. tauri* Bukkva, 1986 from domestic cattle *Bos taurus* Linnaeus, 1758. However, *D. tauri* is smaller, differs with regard to the shape of the gnathosoma (*D. tauri* – rectangular, length less than width at the base; *D. bialoviensis* sp. nov. – square with width close to length, if rectangular, length greater than width at base), and important taxonomic features, such as the location and shape of supracoxal spines (*T. tauri* – smaller, spatulate), spines on palpi (*D. tauri* – 2 similar, forked and one single), and the subgnathosomal setae (*D. tauri* – elongated structures located on both sides of the middle part of the pharyngeal bulb are visible); the shape of epimeral plates is also different, especially the IV pair of the female, only being slightly arched at the posterior edges; the shape of the aedeagus (straight, narrower, located a slightly lower). In addition, its typical microhabitat is the skin around the eyes.

## 4 Discussion

Taking into consideration the whole set of studied morphological features, *Demodex bialoviensis* sp. nov. from European bison appears significantly different from all known Demodecidae. In bison, it also occupies a specific microhabitat, which is the skin of the nasal region.

The Demodecidae are characterized by topical specificity, i.e., occupying a strictly defined microhabitat (e.g. follicles of normal hair, sensory hair, sebaceous glands, modified glandular organs, epidermis, auditory canals, tissues of the tongue), or topographic preference, i.e. a preference for a body region, such as the head (Izdebska and Rolbiecki, 2013). Other species are known to inhabit the nasal region, such as D. ratticola Bukva, 1995 from the brown rat *Rattus norvegicus* (Berkenhout, 1769) (see Bukva, 1995; Izdebska and Rolbiecki 2014), or in ungulates, *Demodex* sp. is found on the red deer *Cervus elaphus* Linnaeus, 1758 (see Bukva and Preisler, 1988; Izdebska and Fryderyk, 2012). A good point of reference for the present case is undoubtedly the deer *Demodex* sp., which also shows relatively low infestation parameters: a prevalence of 25% and density of 1.5 per 1 cm^2^ has been noted in studies from Poland (Izdebska and Fryderyk, 2012). Similarly, *D. bialoviensis* sp. nov. appears to be a relatively rare species, showing low densities in host skin; the low detection rates may well be associated with that fact that it can occur asymptomatically. Both males and females, and a few juvenile stages, were found in the studied bison. Demodecidae, being stationary parasites, are present in the host throughout the year. However, they may exhibit different population dynamics at different times. Unfortunately, the limited possibility of obtaining material from bison, which are usually studied in winter, makes it impossible to trace the full seasonal population dynamics of these parasitic arthropods. For comparison, *D. bisonianus* demonstrated high infestation parameters (50%) and complete population structure (all life stages) in nearly 400 bison examined during the winter period for more than twenty years (Izdebska, 2001a; 2001b, 2006, 2007, and unpublished data), but it was also recorded in a few hosts collected for analysis during the summer period (J.N. Izdebska, unpublished data).

Typically, a number of specific Demodecidae co-occur in the same host species, with each species occupying different microhabitats. Among ungulates, three specific species have been described in domestic cattle, and two each in sheep *Ovis aries* Linnaeus, 1758, horses *Equus caballus* Linnaeus, 1758, and various wild mammals, including red deer (Izdebska and Rolbiecki, 2020). Among the known Demodecidae, the bison *Demodex* fauna seems to be the most similar to domestic cattle. In both hosts, large (400–500 μm in length), morphologically similar demodecid mites inhabit the Meibomian glands of the eyelids (*D. bisonianus* in bison and *D. ghanensis* Oppong, Lee et Yasin, 1975 in cattle); less hairy skin areas are occupied by medium-sized, slender *D. tauri*, around the eyes of cattle, and the currently-described *D. bialoviensis* sp. nov. in the nasal region. However, the most commonly-recorded demodecid mite species from cattle is *D. bovis*, associated with the hairy skin of the body (Izdebska, 2009; Izdebska and Rolbiecki, 2020), whose counterpart, if it existed, has not yet been discovered or has not survived in the host. Interestingly, an unknown *Demodex* sp. with a similar habitat has been found in the American bison *B. bison* (Linnaeus, 1758), the closest relative to the European bison; however, so far the acarofauna of this mammal has not been studied in more detail and remains virtually unknown.

## Declaration of competing interest

Authors have no conflict of interest to declare.
